# Zero and root loci of disturbed spring–mass systems

**DOI:** 10.1098/rspa.2013.0751

**Published:** 2014-04-08

**Authors:** Christophe Lecomte

**Affiliations:** Southampton Statistical Sciences Research Institute, University of Southampton, Southampton, UK

**Keywords:** particle chain, complex poles, exclusion regions, atomic lattices, bubble vibration, mode veering

## Abstract

Models consisting of chains of particles that are coupled to their neighbours appear in many applications in physics or engineering, such as in the study of dynamics of mono-atomic and multi-atomic lattices, the resonances of crystals with impurities and the response of damaged bladed discs. Analytical properties of the dynamic responses of such disturbed chains of identical springs and masses are presented, including when damping is present. Several remarkable properties in the location of the resonances (poles) and anti-resonances (zeros) of the displacements in the frequency domain are presented and proved. In particular, it is shown that there exists an elliptical region in the frequency–disturbance magnitude plane from which zeros are excluded and the discrete values of the frequency and disturbance at which double poles occur are identified. A particular focus is on a local disturbance, such as when a spring or damper is modified at or between the first and last masses. It is demonstrated how, notably through normalization, the techniques and results of the paper apply to a broad category of more complex systems in physics, chemistry and engineering.

## 1. Introduction

The dynamics of collinear chains of particles has been studied for a long time. Although apparently simple, these systems exhibit remarkable properties. Their interest is not limited to the academic realm as they model the fundamental vibrations of crystals in solid-state physics [1,2], the atomic and molecular dynamics of chains of molecules in physics, chemistry and biology [3–7], as well as the behaviour of real-life objects such as structures with repetitive components or rods and beams that are widely used in engineering. They are directly related to linear algebra in which they are represented by particular matrices, such as tri-diagonal, multi-diagonal, circulant or Toeplitz matrices, as well as to the theory of orthogonal polynomials. They are also often used to study or illustrate theoretical aspects like the effect of disorder on systems [8,9]. Their properties indicate how the systems can behave when used within a controlled system or when some of their components are perturbed or damaged, for example in the case of damaged bladed discs or impurities in crystals [10–15].

However simple these systems appear, however important they are and for however long they have been studied, they are still under investigation. The present contribution is notably closely related to recent work on analytical expressions of the eigenvalues, eigenvectors and inverse of tri-diagonal matrices that have two or four of their corner coefficients disturbed [16–21]. The focus here is on the location of not only poles but also zeros of the transfer functions of a disturbed system in the frequency–disturbance magnitude plane. These loci are important system properties as they notably indicate if a system is stable, observable or controllable [22–24]. The loci of zeros also allow the localization of stable and unbiased frequency points in the theory of uncertain dynamic systems [13,25]. Analytical explicit expressions of the transfer functions of the system are derived by making use of Chebyshev polynomials and low-rank updates.

After presentation of the system and resulting equations of motion in §2, the document is mainly split into the study of the nominal or undisturbed system, and that of the system with rank-one disturbance. For both, the exact expressions of all transfer functions are first presented in §3. Properties of the nominal system are then presented in §4. This includes the explicit expressions for the locations of poles and zeros of the transfer functions. Similar results and properties are presented in §5 for the disturbed system. Along the way, remarkable properties of the transfer function from the first to last mass are presented. In the case of the nominal system, it is shown in §4*c* that, besides the known property that this transfer function has no zero, its magnitude is also excluded from a circular region. Similarly, for the disturbed system, it is shown in §5*b*(i) that no zeros of this transfer function exist in an elliptical region of the real plane defined by the frequency parameter and the disturbance magnitude. Other exclusion and inclusion properties for the loci of poles and zeros are presented, such as the location of multiple poles of the disturbed system in §5*a*(ii).

## 2. Nominal and disturbed spring–mass systems

**Figure 1.**
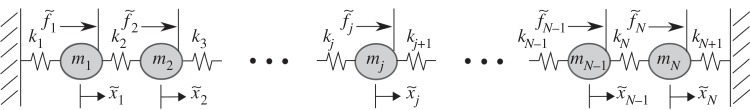
Illustration of the nominal collinear spring–mass systems for *N* masses.

Considered is a nominal collinear system of alternating springs and masses, as illustrated in figure 1. The first and the last components of the system are springs that are connected to fixed points.

### (a) Nominal spring–mass systems

From Newton's second law, the equations of motion of the masses are easily found to be
2.1
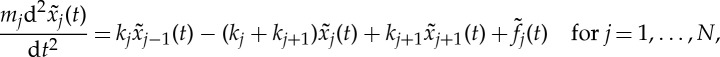
where *k*_*j*_ denotes the spring constants, *m*_*j*_ denotes the masses, and 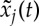 and 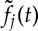 are, respectively, the displacements of the masses and the external forces as a function of time, *t*. Note that 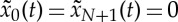 is defined for conciseness. The Fourier transform gives the frequency domain equations that may be expressed in compact matrix form as 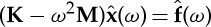 with diagonal mass matrix **M**, tri-diagonal stiffness matrix **K**, and input and output vectors 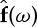 and 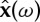, respectively.

The nominal system considered here has all its masses, as well as all its spring constants, equal to each other, *k*_*j*_=*k*,*j*=1,…,*N*+1 and *m*_*j*_=*m*, *j*=1,…,*N*. Based on this assumption, the matrix **K** is *k* times the tri-diagonal matrix **T**, which has 2's on its diagonal and −1's on its two neighbour diagonals, while the matrix **M** is *m* times the identity matrix **I**. It is also assumed that all the components of the force vector have the same time dependency so that one can work with the constant vector, 
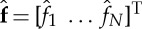
. This corresponds to an impulse force, 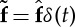, where *δ*(*t*) is the Dirac delta function. In matrix form, the nominal equations of motion are therefore
2.2
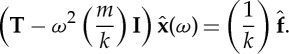


The normalized case *k*=*m*=1 covers a wide range of cases. For example, the present case can be expressed with the normalized frequency parameter, *λ*=*ω*^2^(*m*/*k*), force vector, 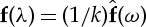, and output vectors, 
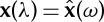
. There is also no restriction to undamped systems, because the generally complex frequency parameter, *λ*, permits the consideration of damping.

### (b) Disturbed spring–mass systems

**Figure 2.**
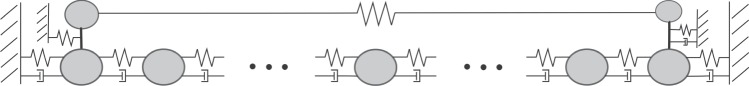
Illustration of a regular collinear system disturbed with components added to the pair of first and last masses.

Some disturbance is allowed to the nominal spring–mass systems. Specifically, a different impedance is considered for the subsystem made of the two extreme (first and last) masses, as illustrated in figure 2. This disturbance might, for example, be an additional spring connecting the two masses, a small mass added on the first mass, or an additional spring connecting the last mass to a fixed point. Any such disturbance is *a priori* allowed (one does not prescribe the disturbance to necessarily be a combination of springs or masses or even physically possible). In order to conveniently study its effect on the nominal system, it is expressed as the product of a unit disturbance scaled by a scalar, *s*. In matrix form, this results in the bi-parameter system 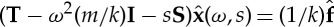, where the three normalized matrices are
2.3
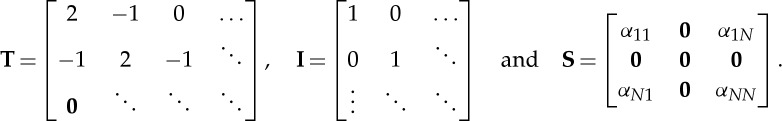
As in §2*a*, the normalized vectors are 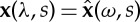 and 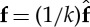 for *λ*=*ω*^2^(*m*/*k*). For simplicity, **S** is rank-one, i.e. it may be expressed as the outer product of two vectors, 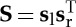.

### (c) Normalized spring–mass systems

It was seen in §2*a*,*b* that the equations of motion can be written in a normalized form that is equivalent to working with unit mass and stiffness, and with a frequency parameter, λ=*ω*^2^. The same normalized systems, (**T**−λ**I**)**x**(λ,0)=**f** and (**T**−λ**I**−*s***S**)**x**(λ,*s*)=**f**, are studied in the rest of the paper. They support a wide range of situations as illustrated in the following subsections. Note the mnemonic notation, **T** for *t*ri-diagonal, **I** for *i*dentity and **S** for *s*quare.

#### (i) First application: normalized damped system

**Figure 3.**
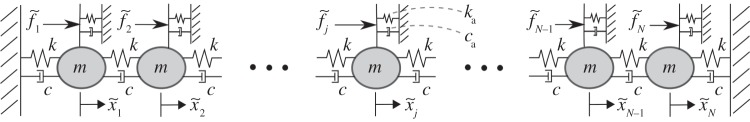
Illustration of a regular damped collinear spring–mass system.

A first normalization that involves damping is now illustrated with the *damped benchmark* problem schematized in figure 3. The nominal equations of motion are found to be
2.4
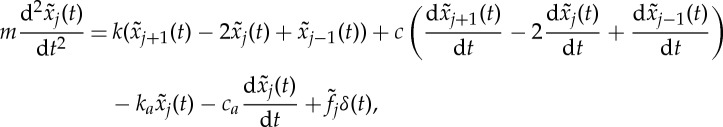
where *c* is the additional damping coefficient and *k*_a_ and *c*_a_ are the real positive stiffness and damping coefficients of the additional local elements. This results in the nominal matrix system
2.5
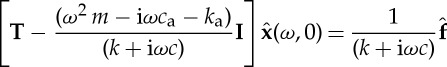
with complex frequency argument λ=(*ω*^2^ *m*−i*ωc*_a_−*k*_a_)/(*k*+i*ωc*) and normalized force 
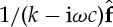
. Similarly, considering the disturbance leads to a non-normalized system such that
2.6
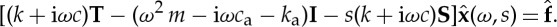

*Particular case.* Equation (2.4) may appear in many contexts. It can, for example, describe the linearized behaviour of nonlinear systems around an equilibrium or solution point. Particular cases of such a situation include that of the linearized discrete form of the sine-Gordon equation 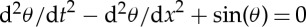 that notably appears in the modelling of magnetic flux transmission of Josephson-junction transmission lines [26]. The poles of this linearized sine-Gordon equation around soliton solutions could then provide information about the spectrum of the phonons and the corresponding eigenmodes would be the phonons far away from the solitons [27,28]. Considering equation (2.4) and disturbed spring–mass systems, such analysis could possibly involve forces, damping and disturbance.

#### (ii) Second application: normalized multi-degree-of-freedom system

**Figure 4.**
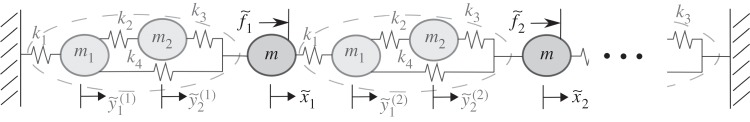
Illustration of a regular collinear system with multi-d.f. springs.

The second normalization example and benchmark application involve non-normalized springs that are multi-degree-of-freedom (multi-d.f.) components. The particular model considered is schematized in figure 4. Each consecutive pair of the *N* masses is separated by an equivalent or generalized spring that consists of two masses, *m*_1_ and *m*_2_, and four springs, *k*_1_–*k*_4_. The full system has therefore more than *N* degrees of freedom. The transformation into the normalized form starts with the resolution of the equations of motion inside individual spring components. Looking specifically at the component between the (*j*)th and (*j*+1)th masses and at mass *j* gives
2.7
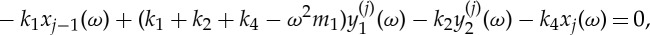

2.8
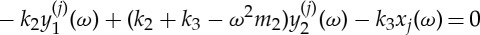

and
2.9
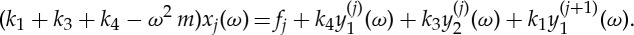
Using equations (2.8) and (2.7), the internal variables, 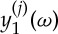 and 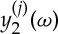, can be solved in terms of the neighbouring variables, *x*_*j*−1_ and *x*_*j*_, and substituted in equation (2.9). Basic algebra gives, for identical *m*_1_=*m*_2_=*m*_s_ and *k*_1_=⋯=*k*_4_=*k*_s_, and with *ω*_s_^2^=*k*_s_/*m*_s_,
2.10
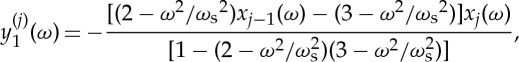

2.11
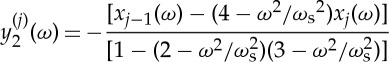

and
2.12
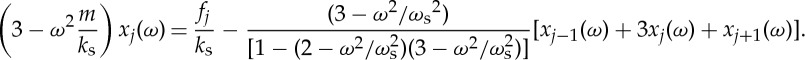
Although it may not be obvious, this equation can easily be written up in the normalized form by two operations: the multiplication of both sides by 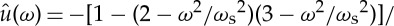 (3−*ω*^2^/*ω*_s_^2^), and the addition and substraction of 5*x*_*j*_(*ω*) from the bracket [*x*_*j*−1_+3*x*_*j*_+*x*_*j*+1_]=[*x*_*j*−1_−2*x*_*j*_+*x*_*j*+1_]+5*x*_*j*_. This results in the desired form (**T**−λ**I**)**x**(λ)=**f***u*(λ), where 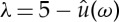 (3−*ω*^2^*m*/*k*_s_) is a rational function of *ω*^2^ with cubic numerator and linear denominator. For each value of λ equal to a pole or zero of the normalized system, it is thus possible to retrieve the corresponding values of *ω*^2^ by trivially solving a cubic polynomial.

Some care must now be taken with the right-hand side, i.e. with the normalized vector, **f***u*(λ). Its scalar magnitude can indeed be zero or infinite for discrete values of *ω*^2^ that cancel the quadratic numerator or linear denominator of *u*(λ). Possible simultaneous cancelling of the numerator and denominator must be carefully considered as well as the *a priori* possible cases where a pole or zero of the system (**T**−λ**C**) is neutralized, respectively, by a zero or pole of the magnitude, *u*(λ). With these considerations, the zeros and roots of the normalized system and of the scalar function *u* provide all those of the non-normalized system. Note that in the case of the first benchmark, i.e. in equation (2.5), the frequency-dependent scaling function 1/(*k*+i*ωc*) can be neither zero nor infinite as long as the real *k* and *c* are strictly positive. All zeros and poles of the non-normalized and normalized systems (for a constant force) are therefore identical.

For the current multi-d.f. benchmark, special attention must also be paid to the additional internal d.f. In order to avoid missing internal modes, the poles of the internal systems—in the present case, the roots of the polynomial 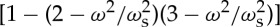
—must be analysed. Finally, the study of the transfer functions and zeros of the normalized system provides direct information only for the main masses of the whole original system. More information is however available. For example, equations such as (2.10) and (2.11) provide all the system responses from those at the main masses. These expressions can thus be used to carry out the work of identification of zeros at the internal masses. By reciprocity, the unit transfer functions from an internal mass to a main mass can also be treated in a dual manner.

**Figure 5.**
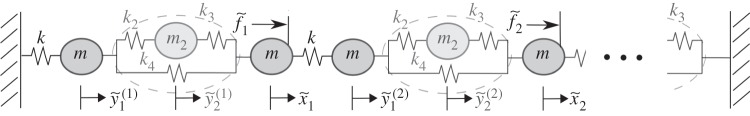
Illustration of a colinear spring–mass systems with alternating regular and one-degree-of-freedom springs.

*Other cases.* Under the same umbrella as that of the current benchmark, multi-d.f. systems such as in figure 4 allow one to deal directly with other systems such as the one illustrated in figure 5, where a modified stiffness component (the 1 d.f. subsystem circled by a dashed line) only appears every other spring. In this case, this is simply done by using *k*_1_=*k* and *m*_1_=*m*. The case of diatomic or multi-atomic lattice vibrations [2] is also covered by this benchmark, for both alternating masses and alternating springs. Periodic composite materials such as those described in Silva [29] and Andrianov *et al*. [30] can be put in the same framework. Furthermore, the present approach can be directly applied to condense macro cells of a homogeneous chain, when considering a so-called multi-field approach of vibrations as in Vasiliev *et al*. [31]. This can be illustrated by the case of figure 4 with *m*_1_=*m*_2_=*m*, *k*_1_=*k*_2_=*k*_3_=*k* and *k*_4_=0.

#### (iii) Third application: bubbles vibrating in an acoustic field

The third application concerns the case of gas bubbles in water vibrating in an acoustic field. The dynamics of such bubbles is a very complex and important field of study, as its behaviour is highly nonlinear for large radial changes [32] so that it can lead to locally extreme conditions of velocity, pressure and temperature. The study of the linear vibrations of the bubble surfaces is also very important as their acoustic response, when submitted to an acoustic field, can be used as a tool to identify and count individual bubbles from a gas leakage [33]. In the presence of several bubbles, there is interaction and resonance because of coupling through radiated acoustic pressure from one bubble to the other. This effect can be modelled by this theory for the bubble vibrations in their linear regime.

The corresponding normalization is demonstrated here by starting from the coupled linearized equations of motion of two identical bubbles [34–36]
2.13
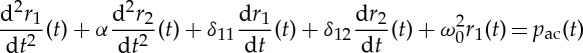
and
2.14
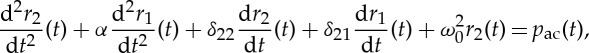
where *r*_*j*_ denotes the variation of the radius *R*_*j*_ of the *j*th bubble compared with its equilibrium value, *R*_0_, i.e. *R*_*j*_(*t*)=*R*_0_+*r*_*j*_(*t*); *α*=*R*_0_/*d* is the ratio of this equilibrium and the distance, *d*, between the two bubbles; *p*_ac_ is a force acting on the system that is proportional to the incident acoustic field in which the bubbles are residing; and *ω*_0_ is the natural Minnaert's frequency of resonance of the bubbles. The factors *δ*_*jk*_ are coupled damping coefficients that are here chosen by symmetry, such that *δ*_12_=*δ*_21_=*δ*_*c*_ and *δ*_22_=*δ*_11_=*δ*_a_.

The first step to normalization is the premultiplication of the system, expressed in matrix form, by the inverse of the matrix 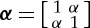. In the frequency domain, this gives
2.15
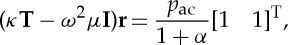
where the frequency-dependent equivalent stiffness and mass of the bubbles are 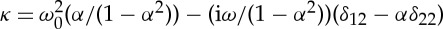 and 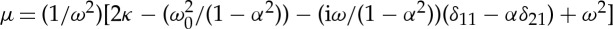. The division of both sides of this equation by *κ* then provides the normalized equation with λ=*ω*^2^*μ*/*κ* and right-hand side **f**=[1 1]^*T*^*p*_ac_/[*κ*(1+*α*)].

All analysis in this paper can therefore be directly applied to analyse the vibration of gas bubbles in liquid. In particular, all the results about the transfer functions, *g*_1*N*_, from the first to the last mass correspond to the transfer functions between the two bubbles in the present case, *N*=2. Note that even though the boundary conditions of the bubbles might not formally be equivalent rigid attachments, this model can perfectly be used, without any approximation or error, to analyse the two bubbles. One advantage of the theoretical model of this paper is that otherwise difficult to measure properties of the bubble–fluid system can be evaluated: by matching the predicted loci of the resonances and zeros to their evaluations obtained in a well-controlled experimental set-up, the actual values of parameters such as damping can be appropriately tuned. In the context of identification of gas leaks from pipes, natural methane seeps, or from undersea carbon capture and storage facilities [33], one can also push the analysis further to assess how the cross-bubble coupling affects the acoustic scattering from an ensemble of bubbles and how the consideration or non-consideration of this coupling impacts the measurements of the number of bubbles and quantity of leaking gas. The vibrational behaviour of pairs of bubbles with regard to that of single bubbles is also important in shock wave lithotripsy [37] as the source image of a bubble close to the surface of a kidney stone is exposed to a symmetric source image.

**Figure 6.**
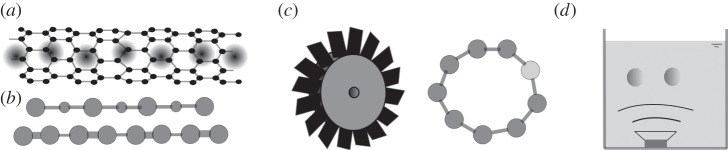
Schematic of some fields of application of this work: (*a*) chain of molecules in a single-walled nano-tube; (*b*) vibrating diatomic lattice with alternating springs or alternating masses; (*c*) vibration of a bladed disc and its simplified model as a circular vibrating system; and (*d*) acoustically excited gas bubble in liquid.

*General applicability.* As illustrated in the previous sections and in figure 6, the work presented here can generally be applied and extended to various fields and the d.f. are not restricted to displacements. They may, for example, be rotations of a beam or values of the acoustic pressure at different points of a waveguide. Note that figure 6*c* corresponds to a circular chain with damage whose transfer functions have been studied in [13], section 5, appendix D, starting from this theory.

## 3. Exact expressions of the nominal and disturbed transfer functions

Considered in this section are the responses or *transfer functions* between a unit force applied at the *k*th mass and the displacement of the *j*th mass. Such transfer functions are denoted
3.1
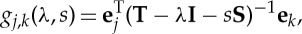
where **e**_*j*_ is a unit vector with only non-zero coefficient at position *j*, in the general, *disturbed*, case while the *nominal* transfer functions in the particular case of absence of disturbance are
3.2
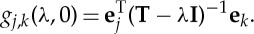
The transfer functions for general input and output vectors, **f** and **c**, are available from linear combinations of those as 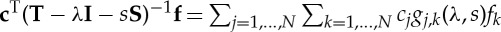. Both the nominal and disturbed transfer functions exhibit remarkable characteristics that are presented further in the paper. Exact expressions of the transfer functions are first derived in §3*b*,*c*, by making use of their link with Chebyshev polynomials which is first explained in §3*a*.

### (a) Transfer functions and Chebyshev recurrence relations

The nominal transfer functions have been studied at least since the time of Rayleigh. They have interesting properties by themselves because of the structure of **T**, which, for a fixed value of *N* and *k*, and even with (fixed) non-zero disturbance, has the inherent recurrence relation of Chebyshev polynomials, presented here in the general case,
3.3
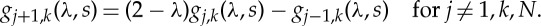
Such recurrence relations are standard in the generation of orthogonal polynomials (e.g. [38]). They are in particular the recurrence relations for the Chebyshev polynomials of second kind, *U*_*n*_(*x*), which are defined by
3.4
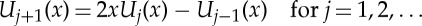
together with the initial functions, *U*_0_(*x*)=1 and *U*_1_(*x*)=2*x*. An alternative trigonometric form of these polynomials is [38], eqns (22.3.16)
3.5
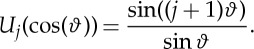
Note that equation (3.3) differs only from equation (3.4) by a shifting and scaling of the argument (*x*=1−λ/2). This is the same kind of operation as the shift used to obtain the shifted Chebyshev polynomials (e.g. [39], p. 179). The recurrence relations and the definitions of Chebyshev polynomials allow easy derivation of the transfer functions, as presented in the next sections.

### (b) Explicit expression of the nominal transfer functions

Explicit expressions of nominal transfer functions are summarized in the following lemma.

**Lemma 3.1**

*Consider the tri-diagonal dynamic system*
3.6
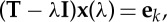
*where* **T** *is the tri-diagonal matrix of dimension *N* with all twos on its diagonal and all negative ones on its neighbour diagonals,* **I** *is the identity matrix of dimension* *N* *and* **e**_*k*_ *is the *k*th unit vector*.

*Then, the components*, *g*_*j*,*k*_(*λ*,0), *of the response*, ***x***(λ)=[*g*_1,*k*_(λ,0)…*g*_*N*,*k*_(λ,0)]^T^, *are*
3.7
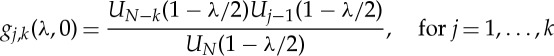
*and*
3.8
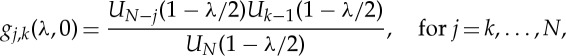
*where the function* *U*_*j*_(*x*) *is the Chebyshev polynomial of second kind of order* *j* (*U*_0_(*x*)=1,*U*_1_(*x*)=2*x*,*U*_*j*+1_(*x*)=2*xU*_*j*_(*x*)−*U*_*j*−1_(*x*), *for* *j*=1,…). *By symmetry*, *g*_*j*,*k*_(λ,0)=*g*_*k*,*j*_(λ,0) *and* *g*_*N*+1−*j*,*N*+1−*k*_(λ,0)=*g*_*j*,*k*_(λ,0) *for all* λ,*N*,*j*,*k*.

**Proof.**

For *s*=0 and fixed values of *N* and *k*, one uses the notation *x*_*j*_(λ)=*g*_*j*,*k*_(λ,0) for compactness. The recurrence relations above and below row *k* of equation (3.6) give
3.9
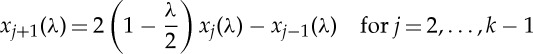
and
3.10
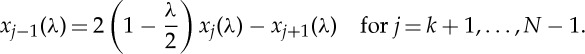
Considering first 2≤*k*≤*N*−1, the first and last rows of equation (3.6) may be written *x*_2_(λ)=2(1−λ/2)*x*_1_(λ) and *x*_*N*−1_(λ)=2(1−λ/2)*x*_*N*_(λ). Along the recurrence relations, they give
3.11
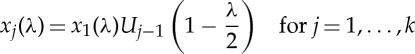
and
3.12
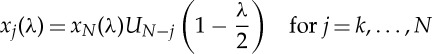
and, in particular, the expressions of *x*_1_ and *x*_*N*_ in terms of *x*_*k*_, *x*_1_(λ)=*x*_*k*_(λ)[*U*_*k*−1_(1−λ/2)]^−1^ and *x*_*N*_(λ)=*x*_*k*_(λ)[*U*_*N*−*k*_(1−λ/2)]^−1^. Substituting these in equations (3.11) and (3.12), at *j*=*k*−1 and *j*=*k*+1, respectively, and carrying the results in the *k*th row of equation (3.6), gives
3.13
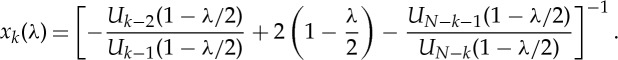
Grouping the two first terms in brackets on the right-hand side of this equation and using the recurrence relation of Chebyshev polynomials results in
3.14
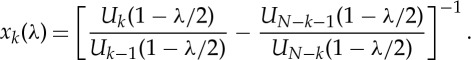
Combining the two terms in brackets on the right-hand side of this equation, considering the numerator, and using the following relation *k*−1 times (with *q*=*k*,*k*−1,…,2 and *x*=1−λ/2):
3.15
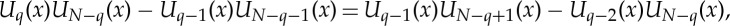
and then the recurrence relation of Chebyshev polynomials a last time, gives
3.16
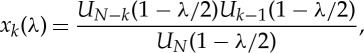
which can then be combined with equations (3.11) and (3.12) to give the expected results for *k*=2,…,*N*−1. Note that equation (3.15) can be obtained by solving for 2*x* in the two occurrences of equation (3.4) obtained for *j*=*N*−*q* and *q*−1, respectively.

For *k*=1, the first row of equation (3.6) and equation (3.12) (which also holds for *k*=1) gives *x*_*N*_(λ)=1/(*U*_*N*_(1−λ/2)) and the expected result. The same approach holds for *k*=*N*. The two symmetry properties can be readily verified from equations (3.7) and (3.8). □

It will be shown in §4 not only that exact expressions of the poles and zeros of the transfer functions can be evaluated from the expressions of the transfer functions but also that their properties can advantageously be studied directly.

Explicit rational expressions of the transfer functions obtained by making use of lemma 3.1 are provided in table 1. While poles and zeros can be evaluated from the expressions of the numerator and denominator polynomials, this becomes unfeasible or unpractical for large dimension systems. Explicit expressions of the poles and zeros will be provided in §4*a*,*b* and will clarify some of their properties. A particular case of the transfer functions from first to last masses that will be further studied is highlighted in the following corollary.

**Table 1.** Samples of the nominal transfer functions from lemma 3.1 for the normalized system.

Dim. *N*	input *k*	output *j*	transfer function *g*_*j*,*k*_(λ,0)	poles	zeros
1	1	1	1/(−λ+2)	2	—
2	1	1	(−λ+2)/(λ^2^−4λ+3)	1,3	2
	1	2	1/(λ^2^−4λ+3)	1,3	—
3	1	1	(λ^2^−4λ+3)/(−λ^3^+6λ^2^−10λ+4)	0.5858,2,3.4142	1,3
	1	2	(−λ+2)/(−λ^3^+6λ^2^−10λ+4)	0.5858,2,3.4142	2
	1	3	1/(−λ^3^+6λ^2^−10λ+4)	0.5858,2,3.4142	—
	2	2	(λ^2^−4λ+4)/(−λ^3^+6λ^2^−10λ+4)	0.5858,2,3.4142	2,2

**Corollary 3.2**

*Equations* (3.7) *and* (3.8) *collapse to* *g*_*k*,1_(λ,0)=*g*_1,*k*_(λ,0)= *U*_*N*−*k*_/*U*_*N*_, *g*_*k*,*N*_(λ,0)=*g*_*N*,*k*_(λ,0)=*U*_*k*−1_/*U*_*N*_, *g*_1,1_(λ,0)=*g*_*N*,*N*_(λ,0)=*U*_*N*−1_/*U*_*N*_, *and* *g*_*N*,1_(λ,0)=*g*_1,*N*_(λ,0)=1/*U*_*N*_ *where, for brevity,* *U*_*j*_ *denotes the value* *U*_*j*_(1−λ/2).

### (c) Explicit expression of the disturbed transfer functions

The following theorem provides the disturbed transfer functions for any values of *s*, *N* and *k*.

**Theorem 3.3**

*Consider the dynamic system* (**T**−λ**I***−s***S**)**g**_*k*_(λ,*s*)=**e**_*k*_*, where* **T** *is the tri-diagonal matrix of dimension N with all twos on its diagonal and all negative ones on its neighbour diagonals,* **I** *is the identity matrix of dimension N,* **S***=***s**_l_**s**^T^_r_ *is a rank one disturbance matrix, such that* **s**_r_*=s*_r1_**e**_1_*+s*_*rN*_**e**_*N*_ *and* **s**_l_*=s*_l1_**e**_1_*+s*_*lN*_**e**_*N*_*, and* **e**_*j*_ *denotes the jth unit vector. Denote α*_*nm*_*=s*_*lm*_*s*_*rn*_ *for n,m=1,N.*

*The components, g*_*j*,*k*_(λ,*s*), *of the response vector,* **g**_*k*_(λ,*s*)=[*g*_1,*k*_(λ,*s*) … *g*_*N*,*k*_(λ,*s*)]^T^*, are
*3.17
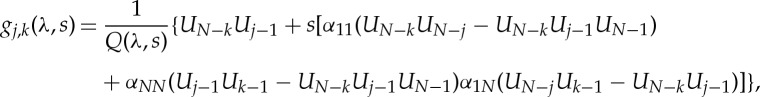
*for j=1,…,k, and
*3.18
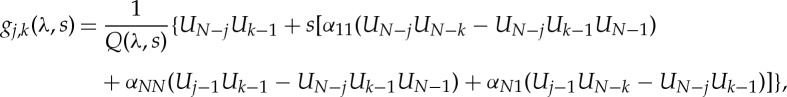
*for j=k,…,N, and, in both cases, Q*(λ,*s*)=*U*_*N*_*−s*[(*α*_11_*+α*_*NN*_)*U*_*N*−1_*+*(*α*_1*N*_*+α*_*N*1_)].

*Here, for brevity, U*_*j*_ *denotes the value U*_*j*_(1−λ/2) *where U*_*j*_(*x*) *is the Chebyshev polynomial of second kind of order j* (*U*_0_(*x*)=1, *U*_1_(*x*)=2*x, U*_*j*+1_(*x*)=2*xU*_*j*_(*x*)−*U*_*j*−1_(*x*), *for j=1,…*).

**Proof.**

The disturbed transfer function may be expressed as a rank-one update of the nominal transfer function (details of this may be found in [13,25]), which gives
3.19
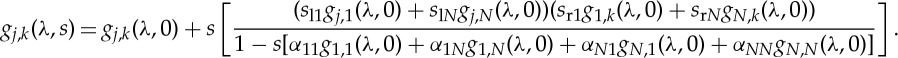
The nominal transfer functions are available from lemma 3.1. For the case 1≤*j*≤*k*≤*N*, one has *g*_*j*,*k*_(λ,0)=*U*_*N*−*k*_*U*_*j*−1_/*U*_*N*_ and the relations of corollary 3.2. Substituting these expressions in equation (3.19) gives equation (3.17). Equation (3.18) is obtained similarly. □

Particular cases appear. If *j*≤*k*, one sees from expression (3.17) that the coefficient of *α*_11_ disappears if *j*=1, while those of *α*_*NN*_ or *α*_1*N*_ disappear if *k*=*N* or *j*=*k*, respectively. Similarly, for *j*≥*k*, *α*_11_, *α*_*NN*_ or *α*_1*N*_ disappear if, respectively, *k*=1, *j*=*N* or *j*=*k*.

## 4. Properties of the nominal transfer functions

The expressions of lemma 3.1 are completely general for any regular collinear system that has identical point elements and connectors between two neighbour elements, independently of their physical nature or the real or complex value of their parameters.

The availability of the exact expression of the responses, particularly in terms of Chebyshev polynomials, allows the identification of properties of the nominal system. Some of these are now presented and then illustrated on the damped benchmark and multi-d.f. problem introduced in §2*c*(i),(ii). A particular focus is on the position of poles and zeros.

### (a) Location of nominal poles

For the poles of the nominal system, one has the following result, which can be, and has been, derived by various methods. Notably, the poles are the eigenvalues of the tri-diagonal matrix **T** and they are the roots of a shifted Chebyshev polynomial [38], eqn 22.16.5.

**Corollary 4.1**

*Using the same notations as in lemma 3.1, for a given value of* *N*, *the poles of any transfer function,* *g*(λ,0)=**c**^T^(**T**−λ**I**)^−1^**f**, *share the same poles which are the roots of the shifted Chebyshev polynomial of second kind and order* *N*, *U*_*N*_(1−λ/2). *In particular, these roots*, 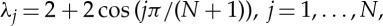 *are real and all located within the finite range* 0<λ<4.

**Proof.**

Trivially, from lemma 3.1, since all *g*(λ,0) are rational polynomials with denominator [*U*_*N*_(1−λ/2)]. The roots, 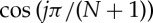, of *U*_*N*_(.) can easily be obtained from equation (3.5). □

The fact that all poles are known and real can be used to derive properties of any particular system that may be expressed as the nominal system. This is illustrated on two applications.

#### (i) Damped benchmark

In the case of the damped benchmark problem, the Chebyshev polynomials have argument
4.1
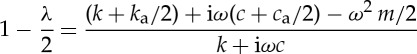

**Figure 7.**
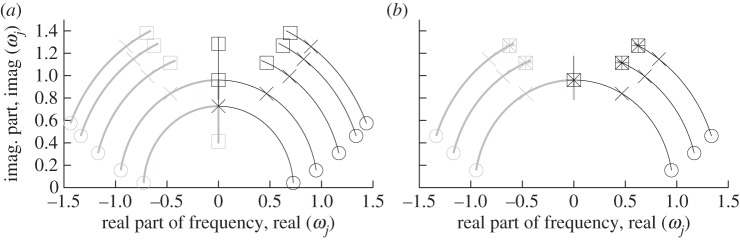
Loci of the (*a*) five pairs of roots and (*b*) three pairs of zeros of the damped benchmark for *N*=5, *k*=0.7, *m*=1.3, *c*=0.4, *k*_a_=0.5 and *c*_a_ varying from 0 to 2.15. The circles, crosses and squares indicate the locations, respectively, for *c*_a_=0 and the two smallest critical values of the poles, *c*_a_=1.7837 and 2.0980, while the stars indicate the location of the zeros at their first critical value, also at *c*_a_=2.0980.

4.2
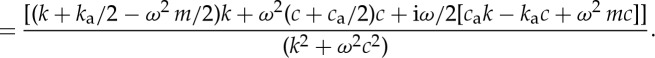
Inverting this relation, the characteristic equation *ω*^2^ *m*−i(*λc*+*c*_a_)*ω*−(*λk*+*k*_a_)=0 follows. There are therefore two possible values of *ω* corresponding to any value of λ,
4.3
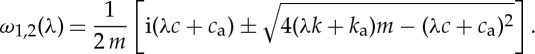
As all poles, in the λ domain, are in the open real interval 0<λ<4 and since (λ_*j*_*k*+*k*_a_)*m*>0, their corresponding values *ω*_1,2_(λ_*j*_) in the *ω* domain have a strictly positive imaginary component as long as *c* or *c*_a_>0. The critical damping, *c* or *c*_a_, can be identified by the value 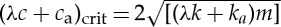 that separates regions where the argument of the square root is positive or negative. When the argument is negative, the *ω*-eigenvalue is overdamped, i.e. purely imaginary so that the corresponding modal component of an impulse response is non-oscillatory in nature. In general, the critical damping values vary from one eigenvalue to another. This is illustrated by the root loci of figure 7 for *N*=5 and varying values of the additional viscous parameter, *c*_a_.

The poles for *c*_a_=0 are *ω*_1,2_(λ_*j*_)={±0.7261+0.0412i,±0.9484+0.1538i,±1.1691+0.3077i,±1.3368+0.4615i,±1.4368+0.5742i}, and the corresponding critical values are *c*_a_={1.7837,2.0980,2.3432,2.4770,2.5302}.

#### (ii) Multi-degree-of-freedom benchmark

Turning to the particular multi-d.f. benchmark, there is a total of 3*N*+2 masses for *N* main masses. For *N*=3 and 11 masses, there are three roots of the normalized system, 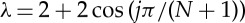 for *j*=1,…,*N*, i.e. λ=0.5858,2.0000,3.4142. Then, solving 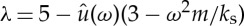 for *ω*^2^ provides nine root values of *ω*^2^. The consideration of the additional roots of the polynomial 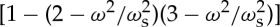, as discussed in §3*b*, provides a supplement of two poles, 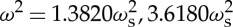, that correspond to λ=5. The last possibility of resonance cancels the denominator of the right-hand side of equation (**T**−λ**I**)**x**=**f***u* at *ω*^2^=3*ω*_s_^2^. For particular values of *m*=2, *m*_s_=3 and *k*_s_=0.7, this corresponds to a degenerate equation with 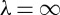. There is no additional resonance as expected, and the whole set of 11 poles is 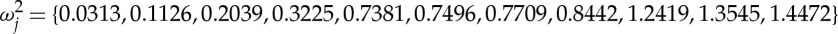.

### (b) Location of nominal zeros

Contrarily to the poles that are identical for any transfer function, the location of zeros varies from one transfer function to the other depending on the particular input and output vectors. The number of zeros itself may vary from as much as *N*−1 to as little as zero. Using lemma 3.1, some properties of the zeros of the mass to mass transfer functions, *g*_*j*,*k*_(λ,0), are now derived.

**Corollary 4.2**

*Using the same notations as in lemma 3.1, for a given value of *N*, the zeros of the transfer functions*, 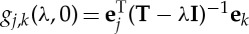, *are the union of the roots of two shifted Chebyshev polynomials of second kind*, *U*_*m*_1__(1−λ/2) *and* *U*_*m*_*N*__(1−λ/2), *of respective orders* 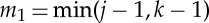 *and* 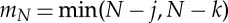. *These zeros*, 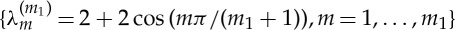 *and* 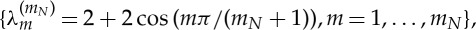 *are real and located within the finite range* 0<λ<4.

**Proof.**

Trivially from the lemma and the evaluation of the roots of the two Chebyshev polynomials at the numerator of the transfer functions. □

The existence and location of the zeros appear to be slightly more elusive than those of the poles. This is particularly true when general input and output vectors, **f** and **c**, are selected. Even if such vectors were real, there would be no insurance that the zeros of the transfer function *g*(λ,0)=**c**^T^(**T**−λ**I**)^−1^**f** would be real. This might seem a contradiction as this transfer function would be a linear combination of real polynomials with real poles and zeros, but is simply explained by the fact that any given real polynomial of order *N*−1 or less may be generated as a real linear combination of the numerator of the nominal transfer functions 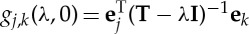, and that polynomials with real coefficients may have complex roots.

The general case does not preclude the existence of properties in particular cases. In corollary 4.2, for unit input and output vectors, one has full information on the existence and location of zeros. This includes the following results.

**Corollary 4.3**

*Corollary* (4.2) *shows that the zeros of the nominal transfer functions* *g*_*j*,*k*_ *have double multiplicity when* *j*−1=*N*−*k*. *Higher multiplicities are impossible*.

**Corollary 4.4**

*Corollary* (4.2) *shows that there is only one finite zero of the transfer function between the first to the penultimate masses of the system. Furthermore, this zero is simply at frequency* λ=2.

As for the case of poles, results of corollary 4.2 can be applied to any system that may be expressed in the form of the nominal system. This allows one to draw conclusions on the location of zeros for a wide range of problems. For example, in the case of the damped benchmark problem, one can use the inverted relation (4.3) and the discussion that follows to prove that all zeros of this problem are complex, with a strictly positive imaginary component as long as *c* or *c*_a_>0.

Additional information may be obtained from Chebyshev polynomial properties.

#### (i) Damped benchmark

In the case of the damped benchmark for *N*=5 and considering the transfer function from the second to the third masses, *g*_2,3_, there are three *a priori* zeros, which are plotted in figure 7, as *m*_1_=1 and *m*_*N*_=2. One can, however, note that each pair of zeros coincides with and therefore cancels a pair of poles. This corresponds, in the case of the first zero, to the value (*j*=4)/(*N*+1), which equals (*j*=2)/(*m*_*N*_+1)=2/3. The cancelling of the poles by a zero in the transfer function between the second and the third masses for *N*=5 is a general property that is independent of the parameter values, *k*,*c*,*k*_a_,…. The critical values at which the zeros have or not a real part are defined by the same equation in terms of λ as that for the critical values of the poles.

While all zeros of all transfer functions from one to another mass coincide with poles in the case *N*=5, its other transfer functions *g*_2,2_,*g*_3,3_,*g*_4,4_,*g*_2,4_,*g*_4,2_ still exhibit actual zeros because of their double multiplicity. The case *N*=5 is somewhat particular, as, in general, not all zeros of unit transfer functions coincide with poles. For example, there is no such coincidence for *N*=4.

#### (ii) Multi-degree-of-freedom benchmark

Considering the multi-d.f. benchmark, again for *N*=3 and a total of 11 masses, all the zeros of the unit transfer functions between the third, sixth and ninth masses are provided by corollary 4.2. The only possible cases where zeros of unit transfer functions between the main masses may exist correspond to non-zero values of *m*_1_ and *m*_*N*_. This can only happen when *j* or *k*=2, i.e. when the second main mass, i.e. the sixth mass, is involved, at the location 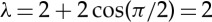. This zero is cancelled by a pole for *g*_1,2_ and *g*_2,3_, so that the only actual zero with double multiplicity is only present in the transfer function *g*_2,2_ at the sixth mass. The additional possibility of zeros between the three main masses has to be considered in the case where the numerator of 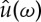 cancels on the right-hand side of (**T**−λ**I**)**x**=**f***u*. For *m*=2, *m*_s_=3 and *k*_s_=0.7, this happens at the locations 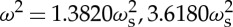 where λ=5. These are actual zeros, as λ=5 is not a root of the normalized system, and the denominator of 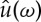 does not cancel at these zeros.

### (c) Magnitude of the nominal transfer functions

The focus of this section is on the particular transfer function from the first to the last masses. As it is the inverse of a Chebyshev polynomial, as presented in corollary 3.2, its properties may be obtained directly from properties of these polynomials. In particular, the following well-known equioscillation property leads to the fact that, in the range of real parameter λ∈[0,4], the transfer function is outside an ellipse.

**Lemma 4.5**

*The scaled Chebyshev polynomial of second kind*, 
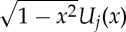
, *equioscillates* *j*+1 *times between* −1 *and* 1 *in the range* *x*∈[−1,1]: **i*t has* (*j*+1) *local extrema in this range and their values alternate between* −1 *and* 1 *for increasing values of* *x*. *The extrema occur at* 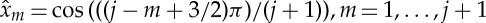 *and the last extremum is a maximum*, 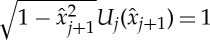.

**Proof.**

In the range *x*∈[−1,1], *θ*=acos(*x*) is real and decreases from *π* to 0. From equation (3.5), one sees that the scaled function 
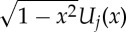
 is 
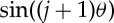
, whose alternating extrema −1 and 1 are reached at (*j*+1)*θ*=(*j*+1/2)*π*,(*j*−1/2)*π*,…,1/2*π*. □

**Figure 8.**
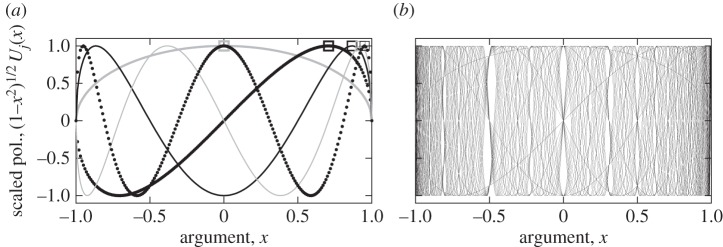
Illustration of scaled Chebyshev polynomials 
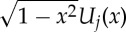
 for *j*=0,…,4 in (*a*), and *j*=0,…,29 in (*b*), as a function of *x*=1−λ/2. The points corresponding to the loci of the last roots are indicated by squares in (*a*).

The scaled Chebyshev polynomials of second kind are presented in figure 8 for the few first orders. The reader intrigued by the ‘white curves’ in this figure is referred to an ‘*asin*–acos’ version of the plots where the curves are straight lines and to Ortiz & Rivlin [40], where this phenomenon is explained. Such properties of the scaled Chebyshev polynomials translate into properties of the transfer functions. Notably, lemma 4.5 allows one to identify properties of the magnitude of the *g*_1,*N*_(λ,0) transfer function as shown in the below corollary of the lemma and corollary 3.2.

**Corollary 4.6**

*Using the same notations as in lemma 3.1, for any real value* λ∈[0,4], *the absolute value of the transfer function*, 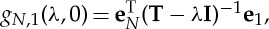 *from the first to the last degree of freedom, is larger than* 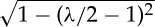. *In other words, in the real plane, all points* (λ,2*g*_*N*,1_(λ,0)) *are outside a circle of radius* 2 *centred at* (2,0). *The transfer function is tangent to this exclusion region at the points* 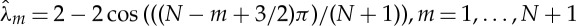. *Also, this transfer function has no finite zero*.

**Figure 9.**
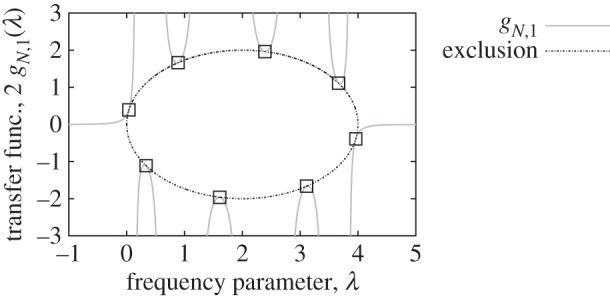
Illustration of the exclusion zone for the values of the nominal transfer function *g*_*N*,1_ for *N*=7. No values (λ,2*g*_*N*,1_(λ)) can lie within the exclusion circle and *g*_*N*,1_(λ) is tangent to this zone at eight points marked by squares.

Although lemma 4.5 is trivial and has been previously noted, as incidentally in [41], property 4.6 of the transfer function appears to not have been identified before. The exclusion zone of the magnitude of the transfer function *g*_*N*,1_ is illustrated in figure 9 for *N*=7. For any other system dimension, *N*, there will be *N*+1 tangency points between the same exclusion zone and the transfer function. They are to be put in the light of those of the scaled Chebyshev polynomial to the constant lines 1 and −1 illustrated in figure 8. Their location at equiangles of the exclusion zone in the λ–2*g*_*N*,1_ plane is evident from their expression in theorem 4.6.

## 5. Properties of the disturbed transfer functions

The properties of the disturbed transfer functions may be derived from their exact expressions given in theorem 3.3. As previously, the focus is now on the location of poles and zeros.

### (a) Location of disturbed poles

The locations of poles of the disturbed transfer functions vary depending on the particular disturbance vectors **d**_*l*_ and **d**_*r*_ but, for any choice of these and a given magnitude, *s*, they are identical for all transfer functions. They are characterized by a necessary zero denominator *Q*(λ,*s*), *Q*(λ,*s*)=*U*_*N*_(1−λ/2)−*s*[(*α*_11_+*α*_*NN*_)*U*_*N*−1_(1−λ/2)+(*α*_1*N*_+*α*_*N*1_)]=0 and are therefore the zeros of a polynomial of order *N* in λ. An alternative point of view is to consider *Q*(λ,*s*) to be a function of *s* (at a fixed value of λ). Two cases may happen: either both *U*_*N*_(1−λ/2) and the coefficient of *s* are zero, in which case any value of *s* corresponds to a pole, or there is just one pole which is the single root of the polynomial of order 1 in *s*. The first case might only happen at a finite number of values of λ as *U*_*N*_(1−λ/2) and [(*α*_11_+*α*_*NN*_)*U*_*N*−1_(1−λ/2)+(*α*_1*N*_+*α*_*N*1_)] are polynomials in λ of order *N* and *N*−1, respectively, which therefore have only *N* and *N*−1 zeros each. An example of the first case is given below. In the second case, the poles are simply defined by the roots, as a rational function of λ. This is expressed in the following theorem, whose proof is trivial.

**Theorem 5.1**

*Using the notations and assumptions of theorem 3.3, for any vectors* **c***,* **f** *and values of α*_*nm*_*'s, all poles of the transfer function* **c**^T^(**T**−λ**I***−s***S**)^−1^**f** *are identified by either the function
*5.1
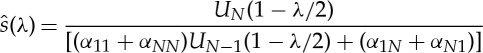
*for any value of* λ, *or any value of s at the discrete values of* λ, *if any, that cancel both the numerator and denominator of expression* (5.1).

The following particular case, which includes additional springs or dampers connecting the first and last massses, is remarkable.

**Theorem 5.2**

*Using the notations and assumptions of theorem* 3.3, *if s*_*r*1_*=−s*_r*N*_ *or s*_*l*1_*=−s*_*lN*_ *then the poles,* 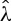
*, of the transfer function g(λ,s)=***c**^T^(**T***−λ***I***−s***S**)^−1^**f** *are equal to
*5.2
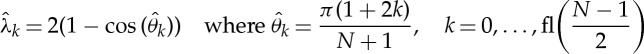
*for any value of s and
*5.3
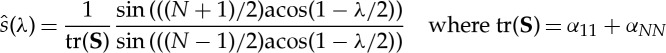
*for any value of* λ. *The ‘floor’ value* fl((*N*−1)/2) *is* (*N*−1)/2 *if it is an integer, or N*/2−1, *otherwise. Note that the value of* 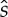 *could be expressed as a ratio of Chebyshev polynomials of second kind with half orders*5.4
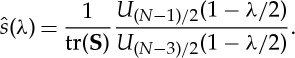

**Proof.**

In either case of equation *s*_*r*1_=−*s*_*rN*_ or *s*_*l*1_=−*s*_*lN*_, one has (*α*_11_+*α*_*NN*_)=−(*α*_1*N*_+*α*_*N*1_). The denominator *Q*(λ,*s*) of the transfer functions with unit input and output vectors described in theorem 3.3 is therefore equal to
5.5
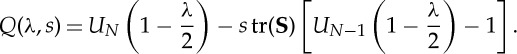
It is a polynomial of strict order *N* in λ and therefore has exactly *N* roots which are the poles of the transfer functions. By definition of the Chebyshev polynomials of second kind, 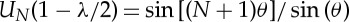 where *θ*=acos(1−λ/2) so that
5.6
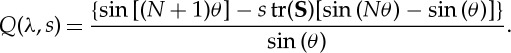
As λ is a function of 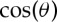, the poles can be identified by the unique values of *θ* in the interval [0,*π*] that cancel the right-hand side of equation (5.6). Particular attention is paid to the values *θ*=0 and *π* as they also cancel the denominator 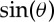. Looking at the numerator, the sinusoidal terms can be developed as [38], eqns(4.3.24/35)
5.7
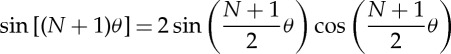
and
5.8
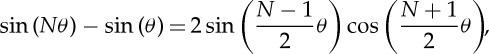
which shows that
5.9
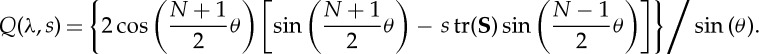
The cosine function is zero for all values 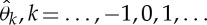. Those, however, only correspond to distinct real zeros, 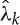, of *Q* for the fl((*N*+1)/2) values, *k*=0,…,fl((*N*−1)/2). This is due to the cyclicity of 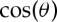 and because the case *k*=*N*/2 for even *N* cancels both the numerator and the denominator in equation (5.9). In the latter case, L'Hospital's rule gives
5.10
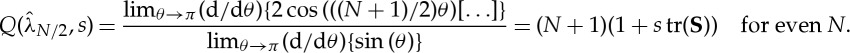
This shows two things, when *N*=2*k* is even: on the one hand, 
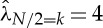
 is not a pole for all values of *s* but, on the other hand, the pair 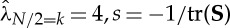 is always a pole—this is a particular case of equation (5.1) for any value *N*.

Now, looking at the term of equation (5.9) in brackets, in the interval 0<*θ*<*π*, each of its roots corresponds to a pole since 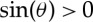 in the selected interval. If both sinusoidal terms in the brackets were zero at the same time, one would have *θ*=2*πk*/(*N*+1) for *k*=1,2,…,fl(*N*/2) at the same time as 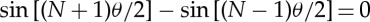, i.e. simultaneously to [38], eqn(4.3.35)
5.11
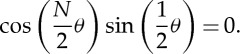
The sine term cannot cancel as its argument is 0<*θ*/2<*π*/2. The cosine term cancels if its argument (*N*/2)*θ*=*πkN*/(*N*+1) is equal to *π*/2+*jπ* where *j*=*kN*/(*N*+1)−1/2 is an integer. This is however impossible since *kN*/(*N*+1)<*k* as long as *N*>−1 and *kN*/(*N*+1)=*k*−*k*/(*N*+1)>*k*−1/2 since *k*≤fl(*N*/2). Therefore, the term in brackets in equation (5.9) can only cancel in the interval 0<*θ*<*π* when
5.12
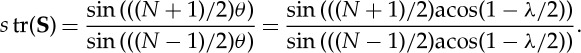
The only values of *θ* that cancel the sine terms and have not yet been considered are *θ*=0 and also *θ*=*π* if *N* is odd. These values however also cancel the denominator in equation (5.9) and the actual non-zero values of *Q*(λ,*s*) are found to be the following by L'Hospital's rule:
5.13
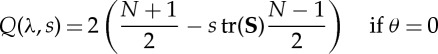
and
5.14
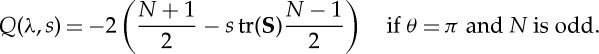
In both cases, it is generally non-zero, except at *s*=[(*N*+1)/(*N*−1)]/tr(**S**), which also corresponds to equation (5.1). All poles have thus been identified. The transfer function with general vectors **c** and **f** has the same poles as those with unit vectors. □

Several general properties of the location of the poles can be derived. These include locations where a pole will always be present and the opposite of regions from which poles are excluded. The location of multiple poles and the real or complex character of the poles are also properties important to know in control. A sample of such properties are proved in §5*a*(i)–(iii). While these results can be directly applied in the situation of any systems that can be normalized, they can also be extended by following strategies similar to those used in the proofs.

#### (i) Fixed location of poles for any system dimension

The following corollary of theorem 5.2 identifies fixed pole locations for any system dimension.

**Corollary 5.3**

*Using the notations and assumptions of theorem* 3.3, *if* *s*_*r*1_=−*s*_r*N*_ *or* *s*_*l*1_=−*s*_l*N*_ *then*

(i) *for any even* *N*=2*k*, *k*=1,2,…, *the pair* 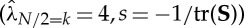 *corresponds to a pole*;
(ii) *for any odd* *N*=2*k*+1, *k*=0,1,…, *the pair* (λ=2,*s*=0) *corresponds to a pole*.

*Furthermore, in either case, these poles are the only ones that are independent of the particular, respectively, even or odd values of *N**.

**Proof.**

That the pair 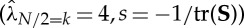 is a pole for any even dimension *N*=2*k* was noted in the proof of theorem 5.2 and it can easily be checked from equation (5.3).

If *N*=2*k*+1 is odd, then corollary 4.1 shows that λ_*j*=*k*+1_=2 is a nominal pole, i.e. it is a pole at *s*=0. Alternatively, two situations can be considered in the odd *N* case, using theorem 5.2. First, if *N*=4 *m*+1 for *m*=0,1,2,…, equation (5.2) with *k*=2 *m* shows that 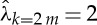 is a pole for any value of *s*, therefore also for *s*=0. Second, if *N*=4 *m*+3 for *m*=0,1,2,…, equation (5.3) shows that 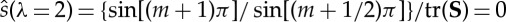.

The unicity of the invariant points for varying even or odd *N* is proved by examining different values of *N*. First, for odd *N*, there is only one possible pole for *N*=1, i.e. 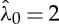, for any *s*. This is a different 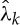 value from those for *N*=3.

**Figure 10.**
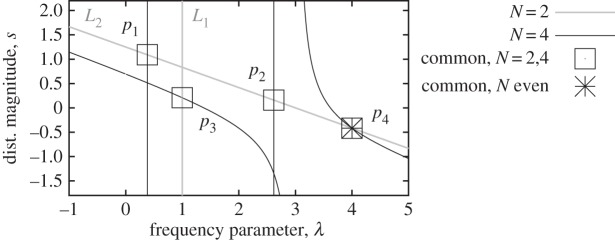
Location of poles in the case of corollary 5.3, for *N*=2,4 and tr(**S**)=2.4. The point *p*_4_ is the only pole common to all even values of *N*.

Second, for even *N* and referring to figure 10, the poles for *N*=2 are along two lines in the real λ,*s* plane: ‘*L*_1_’ such that 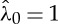 for any *s* and ‘*L*_2_’ such that *s*=1/tr(**S**)(3−λ). (Note that, in order to show the latter, one can apply 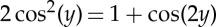 and 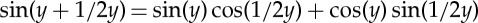 to 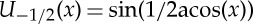 and 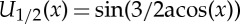. This gives 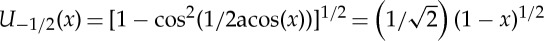 and 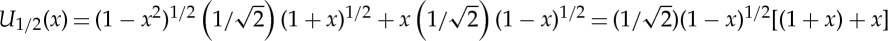. Therefore, *U*_1/2_(*x*)/ *U*_−1/2_(*x*)= 1+2*x* and *U*_1/2_(1−λ/2)/*U*_−1/2_(1−λ/2)=(3−λ).) Now, considering these two lines, one may consider the poles for *N*=4. These happen along three lines: the vertical constant lines, at 
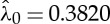
 and 
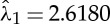
 for any *s* that intersect *L*_2_ at the two points *p*_1_=(λ=0.3820,*s*=2.6180/tr(**S**)) and *p*_2_=(λ=2.6180,*s*=0.3820/tr(**S**)). The line *L*_1_ intersects the curve 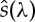 of equation (5.3) for *N*=4 at the point 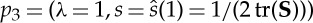 as acos(1−λ/2)=*π*/3 at λ=1. There is a single fourth intersection between the poles at *N*=2 and *N*=4, that is, the fixed point (λ=4,*s*=−1/tr(**S**)). In order to prove this, the expressions of 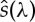 are matched for *N*=2 and *N*=4, which gives
5.15
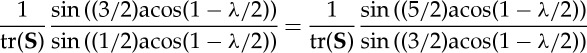

5.16
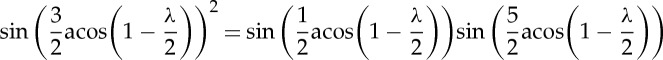

and
5.17
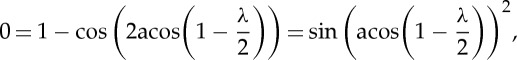
where the trigonometric identity 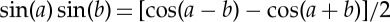 was used to generate the third and the fourth identities. Note that the passage from the first to the second equation through multiplying both sides of the first equation by 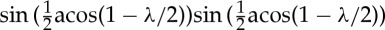 is only valid if this term is non-zero. The fourth equation can be expressed as 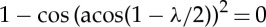, i.e. λ−λ^2^/4=0. This results in two possible common poles: the actual fixed point, *p*_4_=(λ=4,*s*=−1/tr(**S**)), and the possibility λ=0 that has to be discarded as it cancels the denominators of equation (5.15), while L'Hospital's rule shows that its right- and left-hand sides differ. One can easily verify that the three points *p*_1_ to *p*_3_ are not poles in the case *N*=6. □

#### (ii) Location of multiple poles

The frequencies and values of the magnitude disturbance that lead to multiple eigenvalues are important information. In the context of theorem 5.2, the poles can only have multiplicity two at most. The location of the double eigenvalues as well as some of their properties can easily be obtained from the theorem as detailed below.

**Corollary 5.4**

*Using the notations and assumptions of theorem* 3.3, *if* *s*_*r*1_=−*s*_r*N*_ *or* *s*_*l*1_=−*s*_l*N*_ *then*

(i) *the poles have at most multiplicity two*;
(ii) *there are* fl((*N*+1)/2) *poles*, 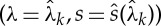 *for* *k*=0,…,fl((*N*−1)/2), *with double multiplicity that are described by equations* (5.2) *and* (5.3);
(iii) *the double multiplicity poles can only be along the two branches of* 1/(tr(**S**)(1−λ/2)) *for* λ *in the open interval* 0<λ<4.

**Proof.**

The two first theses are trivial corollaries of theorem 5.2. In order to prove the third thesis, the expressions of 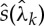 are developed. This gives
5.18
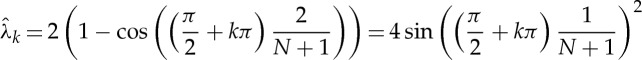
and, using the identities 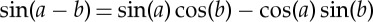 and 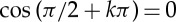,
5.19
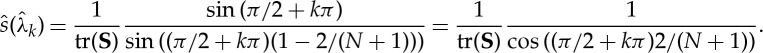
Therefore, 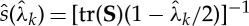. Finally, equation (5.2) shows that 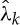 lies in the open interval. □

Damped benchmark.

The identification of the location of the multiple poles is illustrated in the case of the damped system application of §3*a*. Starting from the non-normalized system, equation (2.6), corollary 5.4 can be directly applied provided that the frequency parameter, *ω*, and the disturbance magnitude of interest, *s*′=*s*(*k*+i*ωc*), are properly normalized. For example, if *N*=5, there are only fl((*N*+1)/2)=3 poles with double multiplicity at locations 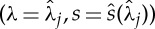 for *j*=0,…,2 with 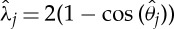, 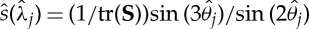, and 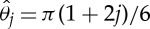. Explicitly, the multiple poles are at the (λ,*s*) locations (0.2679,1.1547/tr(**S**)), 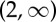 and (3.7321,−1.1547/tr(**S**)). These are consistent with the identified property that 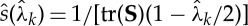 and it is clear that the pole 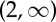 is the only possible multiple pole with infinite value of *s* for any dimension *N*.

**Figure 11.**
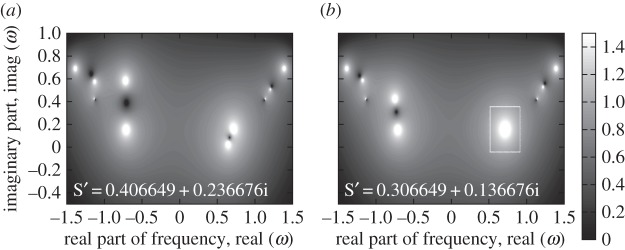
Illustration of multiple pole location. Log_10_ of the absolute value of the transfer function 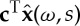 of equation (2.6) for *N*=5. All 10 poles are single in general cases such as for *s*′=0.4066+0.2367i in (*a*) while there is a double pole, which is indicated by the added white box, at *ω*=0.7102+0.1566i for *s*′=0.3066+0.1367i in (*b*). The parameters are *k*=0.7, *m*=1.3, *c*=0.4, *k*_a_=0.5, *c*_a_=0.3, arbitrary random input and output vectors, 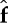 and **c**, **s**_r_(1)=1.5, **s**_r_(*N*)=−1.5, **s**_l_(1)=0.8, **s**_l_(*N*)=−0.8, so that tr(**S**)=2.4.

Two simple operations provide the location of poles in the (*ω*,*s*′) plane: the solution of the following quadratic equation in *ω*, 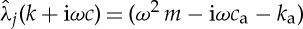 and the further scaling and shifting, *s*′=*s*(*k*+i*ωc*), with the particular solutions 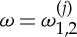:
5.20
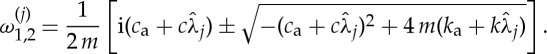
For the particular values *k*=0.7, *m*=1.3, *c*=0.4, *k*_a_=0.5 and *c*_a_=0.3, this results in the four finite poles, (*ω*,*s*)=(±0.7102+0.1566i,(0.7360±0.3280i)/tr(**S**)),(±1.3852+0.6895i,−(0.4898±0.6398i)/tr(**S**)) and in the two poles at *ω*=±1.1325+0.4231i and infinite *s*. The existence of these double poles is illustrated in the maps of figure 11.

#### (iii) Real and complex character of the poles

In the context of theorem 5.2, if the argument λ is real, the value of 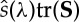 is also real. This might not appear obvious because the arccosine of a real number may be complex. A formal proof of this statement is therefore given and it is incidentally shown that the poles are antisymmetric about λ=2 when *N* is odd. It might be worth noting that there is no (anti-)symmetry of the location of the poles when *N* is even.

**Lemma 5.5**

*Using the same notations and assumptions as in theorem* 5.2, *if* λ *is real, i.e. if* *imag*(λ)=0, *the function* 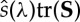 *is real, and*
5.21
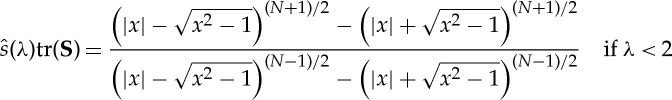

5.22
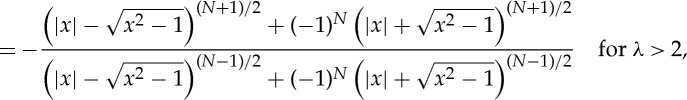
*where* *x*=1−λ/2. *Incidentally, if* λ *is real*, 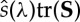 *is antisymmetric about* λ=2 *if* *N* *is odd, i.e.* 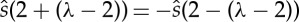 *if *N* is odd*.

**Proof.**

An explicit expression of the complex value acos(*x*) in terms of the logarithm can be derived as follows. One denotes *θ*=acos(*x*)=*θ*_*r*_+*iθ*_*i*_ where *θ*_*r*_ and *θ*_*i*_ are real. As 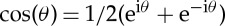 is real its imaginary component 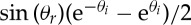 may only be zero if either *θ*_*r*_=*kπ* for an integer *k* or *θ*_*i*_=0. The latter corresponds to acos(*x*) real and values of λ that are in the interval 0≤λ≤4. It will be treated later. The former case corresponds to *θ* complex, real regions |*x*|>1 and intervals λ<0 and λ>4. As the cosine is a cyclic function, there are only two distinct cases, *θ*_*r*_=0 or *θ*_*r*_=*π*, to consider, which, respectively, give 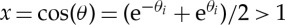 or 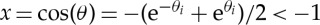. These are quadratic equations in *e*^*θ*_*i*_^: one has either *e*^2*θ*_*i*_^−2*xe*^*θ*_*i*_^+1=0 or *e*^2*θ*_*i*_^+2*xe*^*θ*_*i*_^+1=0 whose roots are, respectively, 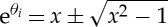 and 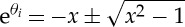. One may only consider one of the two roots in each case as either gives the same ratio of sines in equation (5.12). One chooses the plus sign if *x*>1, λ<0, *θ*_*r*_=0 and the minus sign if *x*<−1, λ>4, *θ*_*r*_=*π*. The imaginary part *θ*_*i*_ of acos(*x*) is obtained by taking the logarithm of the expressions of *e*^*θ*_*i*_^. Thus,
5.23
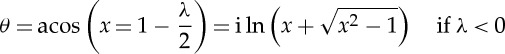

5.24
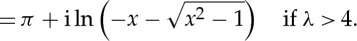
Evaluating the sines of equation (5.12), 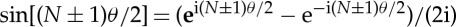 gives, for λ<0, 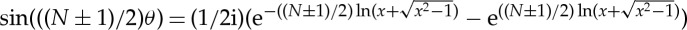. Similarly, by splitting the terms of the exponents, one obtains for λ>4,
5.25
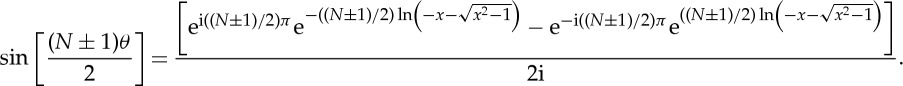
Equation (5.21) is obtained straightforwardly for λ<0 from theorem 5.2 and the fact that 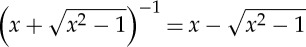 (which can be easily verified). Equation (5.22) is similarly obtained for λ>4 by additionally enumerating the four possible values, *i*,−1,−*i*,1, of e^*i*(*N*+1)*π*/2^ depending on the modulo *m*=*N* mod  4. The expressions are real since |*x*|>1.

The proof for the interior interval −1≤*x*≤1, i.e. for the case *θ*_*i*_=0, follows a similar path. One has 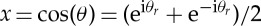 and 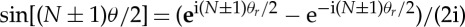. Solving the quadratic equation in e^i*θ*_*r*_^ gives 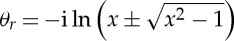, of which a single sign, say negative, needs to be considered and, thus,
5.26
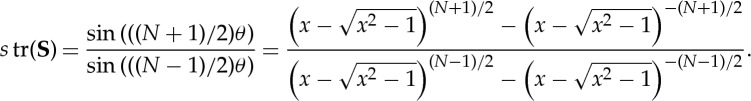
One arrives at equation (5.21) again by using 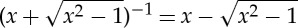 and at equation (5.22) by enumerating the values of (−1)^(*N*+1)/2^ for all values of the modulo *N* mod  4. The expressions are real since 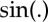 is real for real argument. The antisymmetry is directly verifiable by equation (5.22) and considering that *x*=±(λ−2) at λ=2−(±1)(λ−2). □

The fact that a function *f*(λ) is real when λ is does not automatically imply that the reciprocal is true. It is, however, the case for 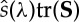 as proved below.

**Corollary 5.6**

*Using the same notations and assumptions as in theorem* 5.2, *if* str(**S**) *is real, then all the poles of the transfer function* *g*(λ,*s*) *occur at real values of* λ.

**Proof.**

The proof proceeds by identifying *N* real poles for any real value of *s* tr(**S**). As *g*(λ,*s*) is a rational function of λ with denominator of order *N* for any fixed *s*, complex poles are then impossible.

The first fl((*N*+1)/2) real poles are independent of *s* and defined by equation (5.2). The remaining fl(*N*/2) real poles for real *s* tr(**S**) are counted by studying the expression of 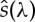 given in equation (5.3). This is done by showing that 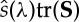 is made of fl(*N*/2) branches that are defined on separate real regions of the frequency parameter λ and such that each has values that vary continuously from 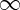 to 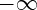.

Looking first at the function of *θ*, 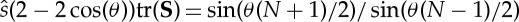, in the interval 0<*θ*<*π* over which 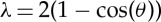 is real, one now shows that its fl(*N*/2−1) poles 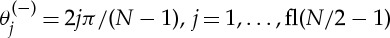 alternate with its zeros 
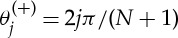
, *j*=1,…,fl(*N*/2): consider a particular pole, 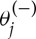, for some integer *j*∈{1,…,fl(*N*/2−1)}. One may express it as 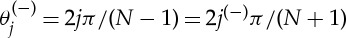 so that the non-integer *j*^(−)^ equals *j*^(−)^=*j*(*N*+1)/(*N*−1). Now, *j*^(−)^>*j* because (*N*−1)/(*N*+1)<1 as long as *N*>−1. One also has that *j*^(−)^<*j*+1 because, if it were not true, one would have *j*(*N*+1)/(*N*−1)≥*j*+1 and therefore 2*j*≥*N*−1 (note that there are no poles of 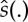 if *N*=1), which is impossible because *j*≤fl(*N*/2−1). As *j*<*j*^(−)^<*j*+1, one has 
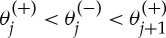
 for all *j*∈{1,2,…,fl(*N*/2−1)}, i.e. this proves that these single poles and zeros strictly alternate in the interval 0<*θ*<*π*.

Consequently, the value of the (λ-)function 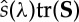 varies continuously from 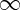 to 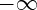 when it goes from any pole to the next in the interval 0<λ<4 and there is a sign change at each pole. The sign change of the value of 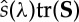 at the poles arises because the poles are single. Similarly, the fact that the zeros are single implies that each branch has to cut the *s*=0 axis only once. The signs at the left and right of each pole may be determined by checking that the value of the rational function at λ=0 is positive. As the function reaches first a zero when λ increases, the function is negative at values of λ slightly smaller than a pole. There are fl(*N*/2−1)−1 branches of the function 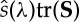 that each vary continuously from 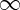 to 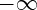 between each of its poles.

One can look again at the poles of the transfer function *g*(λ,*s*) and summarize those that have already been identified in the range of real values 0≤λ≤4. From theorem 5.2, one knows that all poles are on the branches defined by real 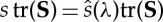 and at 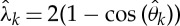 where 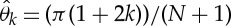, *k*=0,…,fl((*N*−1)/2) for any value of *s*. One already knows that any chosen real value *s* tr(**S**) corresponds to a pole if λ is equal either to one of the fl((*N*−1)/2)+1 real values 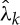 or to one of the fl(*N*/2−1)−1 continuous branches going from one to another pole of 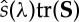. Considering the multiplicity of multiple poles, this corresponds to *N*−2 poles.

As the denominator *Q*(λ,*s*) is a polynomial of order *N* in λ for any value of *s*, there are only *N* poles of the transfer function, *g*(λ,*s*). Its two last poles are below the smallest and above the largest poles of 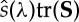: the value of this real continuous function is 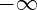 when it reaches its first pole from below. There is a first branch that spans at least all negative values of *s* tr(**S**) and all positive values smaller than or equal to its value (*N*+1)/(*N*−1) at *θ*=0 (otherwise, there would be an excess of poles of *g*(λ,*s*) for *s* tr(**S**)<(*N*+1)/(*N*−1)). Similarly, the last branch spans at least all positive values of *s* tr(**S**). The value of the first branch must therefore be larger than (*N*+1)/(*N*−1) for all λ<0. From equation (5.1) of theorem 5.1, one knows that, as a ratio of polynomials with order of the numerator larger than the order of the denominator, the absolute value of 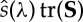 is infinite when λ is infinite. The asymptote of the first (real and continuous) branch is therefore infinity, 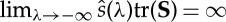. Similarly, 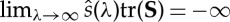. Two additional continuous branches going from 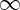 to 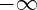 have been identified. For any value of *s* tr(**S**), *N* corresponding distinct (counting multiplicities) real values of λ have therefore also been identified such that the pairs correspond to a pole and there are no other poles. □

### (b) Location of disturbed zeros

The zeros of the disturbed transfer functions vary both for different values of the disturbance parameters (**S** and *s*) and for different input and output vectors. In the case of unit input and output vectors, the exact position of zeros may be found as the zeros of the numerator in the transfer function expressions of theorem 3.3.

**Theorem 5.7**

*Using the notations and assumptions of theorem* 3.3, *the zeros of the transfer function g*_*j*,*k*_(λ,*s*) *cancel the polynomial*
5.27
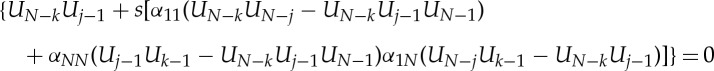
*if j≤k, and the following one, if j≥k*,
5.28
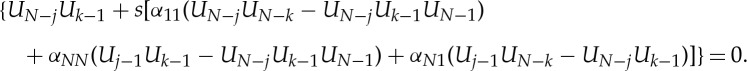

The location of zeros depends in a more or less intricate way on the particular value of **S**. One may identify interesting properties in particular cases.

#### (i) Exclusion region for zeros

**Figure 12.**
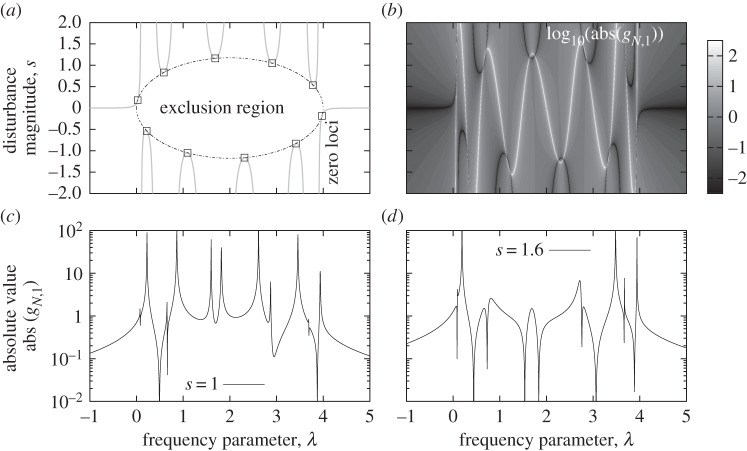
Illustration of the location of zeros of *g*_*N*,1_(λ,*s*) and their exclusion zone. Here, *N*=11, **s**_*l*_(1)=0.3, **s**_*l*_(*N*)=1.7, **s**_*r*_(1)=−0.5, **s**_*r*_(*N*)=0.2. The colour map in (*b*) represents the 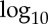 magnitude of the transfer function for various real values of the disturbance magnitude *s*. The location of the (real) zeros matches their prediction from corollary 5.8 as presented in (*a*) where one can note that, as expected, no zeros are present in the exclusion zone. Two particular transfer functions are plotted in (*c*) and (*d*), respectively, for *s*=1 and 1.6. The predicted zeros and poles at real values of λ can be counted in these transfer functions: there are five zeros and 11 poles in the first case, and nine zeros and five poles in the second case.

The following transfer function from the first to last masses is remarkable:
5.29
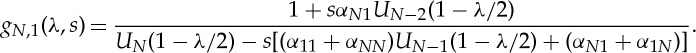
Its zeros are indeed excluded from a region centred at λ=2 for any real argument λ. This property is illustrated in the colourmap of figure 12. The colours in each horizontal line of the colourmap represent the magnitude of the disturbed transfer function for a particular value of the disturbance magnitude, *s*. As illustrated in the figure, the theorems of location of zeros and poles allow one to predict the exact number of real poles and zeros for various values of *s*.

**Corollary 5.8**

*Using the notations and assumptions of theorem* 3.3, *there is no zero of the transfer function* *g*_*N*,1_(λ,*s*) *within a circle* (1/2λ−1)^2^+(*sα*_*N*1_)^2^=1 *of the real* (λ,*sα*_*N*1_) *plane. The curves of zeros are tangent to the circle at pairs of values*, 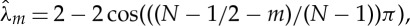 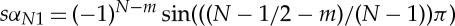 *for* *m*=1,…,*N*−1.

**Proof.**

As the zeros of the transfer function cancel the numerator 1+*sα*_*N*1_*U*_*N*−2_(1− λ/2), they happen at values of λ and *sα*_*N*1_ such that *sα*_*N*1_=*f*(λ), where *f*(λ) is defined as *f*(λ)=−[*U*_*N*−2_(1−λ/2)]^−1^. If λ is real, so is the value of the Chebyshev polynomial *U*_*N*−2_(1−λ/2). One knows from lemma 4.5 that 
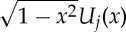
 equioscillates between −1 and 1 in the range *x*∈[−1,1]. One therefore has that 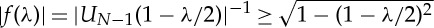 in the range 0≤λ≤4. As *f*(λ) is the value of *sα*_*N*1_ corresponding to a pole for a value λ, the first part of the proof is complete. The location of the tangency points is found from the location of extrema given in the equioscillation lemma 4.5. □

#### (ii) Real and complex character of the zeros

From theorem 5.7 and corollary 5.8, it is clear that not all zeros of the transfer function between the first and the last masses occur at real λ, even if the disturbance *sα*_*N*1_ is real. One can, however, derive properties of the imaginary part of λ at the zeros from the exact expression (5.29). The next result gives exact expression of this imaginary component when the real part of λ is equal to two and *N* is even. It also shows that there is no zero of this transfer function with real *sα*_*N*1_ when real(λ)=2 and *N* is odd. In this case, all zeros correspond to purely imaginary values of *sα*_*N*1_. Incidentally, this shows the corollary that, if *sα*_*N*1_ is a complex number with non-zero real and imaginary parts, there are no zeros of the transfer function with real(λ)=2.

**Theorem 5.9**

*Using the notations and assumptions of theorem* 3.3, *if g*_*N*,1_(λ,*s*)=0 *and* real(λ)=2, *then*
5.30
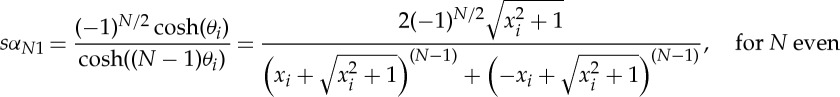

5.31
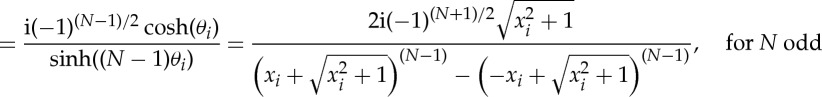
*where both x*_*i*_=(1−λ/2)/i=−1/2imag(λ) *and* 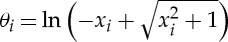 *are real. The hyperbolic sinusoidal functions have their usual definition* 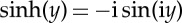 *and* 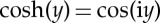.

**Proof.**

All values of λ may be written λ=2−2i*x*_*i*_ for some real *x*_*i*_. Theorem 3.3 and consequent equation (5.29) give that the zeros occur when *sα*_*N*1_=−[*U*_*N*−2_(1−λ/2)]^−1^=−[*U*_*N*−2_(i*x*_*i*_)]^−1^. Sinusoidal expression (3.5) of the Chebyshev polynomials, 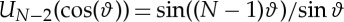, necessitates *θ*=acos(i*x*_*i*_)=*θ*_*r*_+*iθ*_*i*_, where *θ*_*r*_ and *θ*_*i*_ denote real numbers. As
5.32
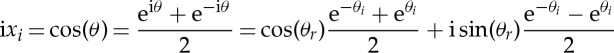
is imaginary, 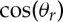 has to cancel, which means that *θ*_*r*_=*π*/2 if 0≤*θ*≤*π* is chosen without loss of generality. Substituting this value gives the quadratic equation in *e*^*θ*_*i*_^, *e*^2*θ*_*i*_^+2*x*_*i*_(*e*^*θ*_*i*_^)−1=0, whose only possible solution is the positive 
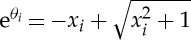
 so that 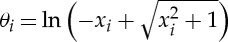. Substituting 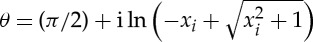 in equation (3.5) and using 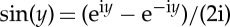 and *e*^*iπ*/2^=*i* completes the proof with little algebra. □

**Figure 13.**
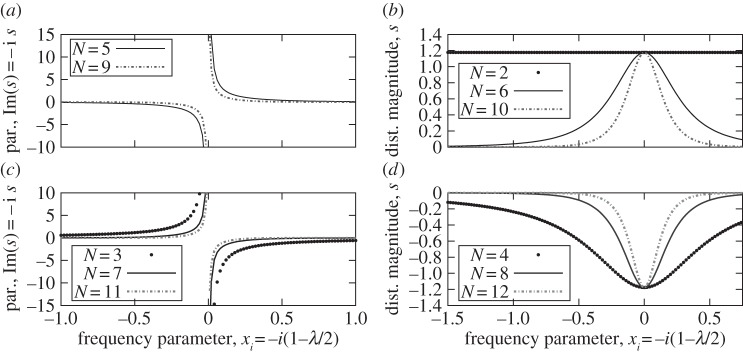
Illustration of the location of zeros of *g*_*N*,1_(λ,*s*) when real(λ)=2, as predicted in theorem 5.9. Depending on the dimension *N*, specifically on the modulo *m*=*N* mod  4, the zeros occur only at purely real or purely imaginary values of *s*. Here, *α*_*N*1_=−0.85. The plots are sorted on the modulo, *m*= (*a*) 1, (*b*) 2, (*c*) 3 and (*d*) 0. There is no zero for *N*=1 and *s*=−1/*α*_*N*1_=1.1765 for *N*=2.

The results of theorem 5.9 are illustrated in figure 13 for a dimension varying from *N*=2 to 12. The fact that a zero occurs at *s*=−1/*α*_*N*1_ for *N*=2 and any value of λ can easily be verified in the expression of the theorem for *m*=2. Also notable is that zeros occur only at real values of *s* for even *N* and at purely imaginary *s* for odd *N*. It is relatively easy to derive additional information about the locations where zeros are absent. For example, the following corollary, whose proof is trivial considering theorem 5.9, is another exclusion result.

**Corollary 5.10**

*Using the notations and assumptions of theorem* 3.3, *if the pair* (λ,*s*) *corresponds to a zero of the disturbed transfer function* *g*_*N*,1_(λ,*s*), *i.e. if* *g*_*N*,1_(λ,*s*)=0 *and if* *sα*_*N*1_ *is a complex number with non-zero real and imaginary parts, then the real part of the* λ *parameter cannot be equal to 2. In other words, if* *g*_*N*,1_(λ,*s*)=0, *the concomitance of the three following conditions is impossible*: real(*sα*_*N*1_)≠0, imag(*sα*_*N*1_)≠0, *and* real(λ)=2.

### (c) Applications of the properties of the disturbed systems

Besides the applications already discussed, two fields in which the current results and approaches about disturbed systems could particularly be used in future work are those of vibration localization [42,43] and mode veering [44–46].

At least two directions may be followed, regarding the analysis of vibration localization, namely the consideration either of the eigenmodes of the disturbed system or of its forced responses. Based on the assessment of localization criteria in these two sets of vectors, such localization could be matched to properties of the systems and disturbance.

The question of mode veering can be addressed from an analytical point of view in the context of particular disturbances considered in this paper. More specifically, as all possible multiple poles have been identified in the case considered in corollary 5.4, the conditions for mode veering or mode crossing are readily available. Mode crossing happens when the mode loci for a particular system and disturbance type go through one of these multiple poles, while mode veering happens when these loci pass closely but not through these particular poles.

## 6. Conclusion

The results presented in this paper about the location of zeros and poles of nominal and disturbed chains of particles are useful at several levels.

First, they can be applied directly to actual physical and engineering systems with repeated particles, bubbles and substructures, possibly after adequate normalization similar to that demonstrated here for damped and multi-d.f. systems and bubble dynamics. The exclusion results ensure that no eigenvalues, zeros or values of the transfer functions can be in given regions of the frequency and disturbance parameters. Such insurance may thus allow one to design structures that are safer and better tolerant to uncertainties and variance in their properties. The identification of the actual locations of zeros, eigenvalues, multiple eigenvalues and pole-zero cancellations of the systems has potential utility in several fields, particularly in, for example, those of control and propagation of uncertainty.

Second, the various techniques used in the paper can be applied and extended to other situations in order to generate further theoretical results. The added or disturbed components can, for example, be considered at other locations than at the two last particles. Three kinds of disturbance may then also be distinguished: the low-rank one as discussed here, the multi-rank case where the disturbance is a sum of several outer products and the more general case where several disturbance magnitudes, *s*_1_,*s*_2_,…, and their associated matrices, **S**_1_,**S**_2_,…, are considered. The location of zeros of transfer functions and their properties can also be studied with different, possibly arbitrary, input and output vectors.

Third, the exact expressions and properties described here can be used as a basis for fundamental characterization and treatment of general dynamic systems. For example, it was explained in the paper that systems with different alternating mass values can be treated as a normalized system. A question is therefore whether there exists a fundamental set of spring–mass systems to which general systems can be reduced. Such a task of categorization also necessitates the consideration of various boundary conditions for the extremities of the spring–mass systems.

Finally, the exact results presented here provide problem cases that can be used as reference benchmarks for various algorithmic techniques to evaluate zeros and roots. For example, approaches to evaluate multiple eigenvalues can be validated by verifying that they can locate the double eigenvalues of the damped benchmark discussed in the paper.

## References

[1] Born M von Kármán Th 1912 On fluctuations in spatial grids. Phys. Z. 13, 297–309

[2] Ashcroft NW Mermin DN 1976 Solid state physics. Toronto, Canada: Thomson Learning

[3] Cvitaš MT Šiber A 2003 Vibrations of a chain of Xe atoms in a groove in a carbon nanotube bundle. Phys. Rev. B 67, 193401 (doi:10.1103/PhysRevB.67.193401)

[4] Matranga C Chen L Smith M Bittner E Karl Johnson J Bockrath B 2003 Trapped CO_2_ in carbon nanotube bundles. J. Phys. Chem. B 107, 12930–12941 (doi:10.1021/jp0364654)

[5] Nishide D 2006 Single-wall carbon nanotubes encaging linear chain C_10_H_2_ polyyne molecules inside. Chem. Phys. Lett. 428, 356–360 (doi:10.1016/j.cplett.2006.07.016)

[6] Wakabayashi T 2007 Resonance Raman spectra of polyyne molecules C_10_H_2_ and C_12_H_2_ in solution. Chem. Phys. Lett. 433, 296–300 (doi:10.1016/j.cplett.2006.11.077)

[7] Malard LM 2007 Resonance Raman study of polyynes encapsulated in single-wall carbon nanotubes. Phys. Rev. B 76, 4 (doi:10.1103/PhysRevB.76.233412)

[8] Allen PB Kelner J 1998 Evolution of a vibrational wave packet on a disordered chain. Am. J. Phys. 66, 497–506 (doi:10.1119/1.18890)

[9] Santos MS Rodrigues ES de Oliveira PMC 1990 Spring–mass chains: theoretical and experimental studies. Am. J. Phys. 58, 923–928 (doi:10.1119/1.16500)

[10] Fang X Tang J Jordan E Murphy KD 2006 Crack induced vibration localization in simplified bladed-disk structures. J. Sound Vib. 291, 395–418 (doi:10.1016/j.jsv.2005.06.020)

[11] Rahimi M Ziaei-Rad S 2010 Uncertainty treatment in forced response calculation of mistuned bladed disk. Math. Comp. Simul. 80, 1746–1757 (doi:10.1016/j.matcom.2009.07.002)

[12] Chan Y-J Ewins DJ The application of robust design strategies on managing the uncertainty and variability issues of the blade mistuning vibration problem. In IUTAM Symposium on the Vibration Analysis of Structures with Uncertainties, St Petersburg, Russia, 5–9 July 2011 (eds Belyaev AK Langley RS), pp. 443–456 IUTAM Bookseries, vol. 27 Dordrecht, The Netherlands: Springer

[13] Lecomte C 2013 Exact statistics of systems with uncertainties: an analytical theory of rank-one stochastic dynamic systems. J. Sound Vib. 332, 2750–2776 (doi:10.1016/j.jsv.2012.12.009)

[14] Maradudin AA 1965 Some effects of point defects on the vibrations of crystal lattices. Rep. Prog. Phys. 28, 331–380 (doi:10.1088/0034-4885/28/1/310)

[15] Lifshitz IM Kosevich AM 1966 The dynamics of a crystal lattice with defects. Rep. Prog. Phys. 29, 217–254 (doi:10.1088/0034-4885/29/1/305)

[16] Bavinck H 1995 On the zeros of certain linear combinations of Chebyshev polynomials. J. Comp. Appl. Math. 65, 19–26 (doi:10.1016/0377-0427(95)00098-4)

[17] Dow M 2003 Explicit inverses of Toeplitz and associated matrices. ANZIAM J. 44, 185

[18] Yueh WC 2005 Eigenvalues of several tridiagonal matrices. Appl. Math. E-Notes 5, 66–74 (http://www.austms.org.au/Publ/Jamsb/V48P1/2347.html)

[19] Kouachi S 2006 Eigenvalues and eigenvectors of tridiagonal matrices. Elec. J. Linear Algebra 15, 115–133

[20] Yueh W-C Cheng SS 2008 Explicit eigenvalues and inverses of tridiagonal Toeplitz matrices with four perturbed corners. ANZIAM J. 49, 361–387 (doi:10.1017/S1446181108000102)

[21] Àlvarez Nodarse R Petronilho J Quintero NR 2012 Spectral properties of certain tridiagonal matrices. Linear Algebra Appl. 436, 682–698 (doi:10.1016/j.laa.2011.07.040)

[22] Gilbert EG 1963 Controllability and observability in multivariable control systems. J. Soc. Ind. Appl. Math. A Control 1, 128–151 (doi:10.1137/0301009)

[23] Kalman R 1965 Irreducible realizations and the degree of a rational matrix. J. Soc. Ind. Appl. Math. 13, 520–544 (doi:10.1137/0113034)

[24] Nagrath IJ Gopal M 2006 Control systems engineering. New Delhi, India: New Age International

[25] Lecomte C Vibration analysis of an ensemble of structures using an exact theory of stochastic linear systems. In IUTAM Symposium on the Vibration Analysis of Structures with Uncertainties, St Petersburg, Russia, 5–9 July 2011 (eds Belyaev AK Langley RS), pp. 301–315 IUTAM Bookseries, vol. 27 Dordrecht, The Netherlands: Springer

[26] McLaughlin DW Scott AC 1978 Perturbation analysis of fluxon dynamics. Phys. Rev. A 18, 1652–1680 (doi:10.1103/PhysRevA.18.1652)

[27] Salerno M Joergensen E Samuelsen MR 1984 Phonons and solitons in the ‘thermal’ sine-Gordon system. Phys. Rev. B 30, 2635–2639 (doi:10.1103/PhysRevB.30.2635)

[28] Dash PC 1986 Defective degenerate mode and dynamics of perturbed sine-Gordon soliton plus phonon. J. Phys. A Math. Gen. 19, 373 (doi:10.1088/0305-4470/19/7/002)

[29] Silva MAG 1991 Study of pass and stop bands of some periodic composites. Acta Acustica united with Acustica 75, 62–68

[30] Andrianov IV Bolshakov VI Danishevs'kyy VV Weichert D 2008 Higher order asymptotic homogenization and wave propagation in periodic composite materials. Proc. R. Soc. A 464, 1181–1201 (doi:10.1098/rspa.2007.0267)

[31] Vasiliev AA Dmitriev SV Miroshnichenko AE 2010 Multi-field approach in mechanics of structural solids. Int. J. Solids Struct. 47, 510–525 (doi:10.1016/j.ijsolstr.2009.10.016)

[32] Keller JB Miksis M 1980 Bubble oscillations of large amplitude. J. Acoust. Soc. Am. 68, 628–633 (doi:10.1121/1.384720)

[33] Leighton TG White PR 2012 Quantification of undersea gas leaks from carbon capture and storage facilities, from pipelines and from methane seeps, by their acoustic emissions. Proc. R. Soc. A 468, 485–510 (doi:10.1098/rspa.2011.0221)

[34] Hsiao P-Y Devaud M Bacri J-C 2001 Acoustic coupling between two air bubbles in water. Eur. Phys. J. E 4, 5–10 (doi:10.1007/s101890170136)

[35] Harkin A Kaper TJ Nadim A 2001 Coupled pulsation and translation of two gas bubbles in a liquid. J. Fluid Mech. 445, 377–411 (doi:10.1017/S0022112001005857)

[36] Ida M 2002 A characteristic frequency of two mutually interacting gas bubbles in an acoustic field. Phys. Lett. A 297, 210–217 (doi:10.1016/S0375-9601(02)00422-X)

[37] Leighton TG Turangan CK Jamaluddin AR Ball GJ White PR 2013 Prediction of far-field acoustic emissions from cavitation clouds during shock wave lithotripsy for development of a clinical device. Proc. R. Soc. A 469, 20120538 (doi:10.1098/rspa.2012.0538)

[38] Abramowitz M Stegun IA 1972 Handbook of mathematical functions. New York, NY: Dover Publications

[39] Lanczos C 1988 Applied analysis. New York, NY: Dover [Reprint. Originally published: 1956 Englewoods Cliffs, NJ: Prentice-Hall]

[40] Ortiz EL Rivlin TJ 1983 Another look at the Chebyshev polynomials. Am. Math. Mon. 90, 3–10 (http://www.jstor.org/stable/2975684)

[41] Borwein P Erdelyi T Zhang J 1994 Chebyshev polynomials and Markov Bernstein type inequalities for rational spaces. J. Lond. Math. Soc. 50, 501–519 (doi:10.1112/jlms/50.3.501)

[42] Pierre C 1987 Localized free and forced vibrations of nearly periodic disordered structures. In. Proc. 28th Structures, Structural Dynamics and Materials Conference, Monterey, CA, 6–8 April 1987. Reston, VA: American Institute of Aeronautics and Astronautics (doi:10.2514/6.1987-774)

[43] Andrianov IV Danishevs'kyy VV Kalamkarov AL 2013 Vibration localization in one-dimensional linear and nonlinear lattices: discrete and continuum models. Nonlinear Dyn. 72, 37–48 (doi:10.1007/s11071-012-0688-4)

[44] Pierre C 1988 Mode localization and eigenvalue loci veering phenomena in disordered structures. J. Sound Vib. 126, 485–502 (doi:10.1016/0022-460X(88)90226-X)

[45] Mace BR Manconi E 2012 Wave motion and dispersion phenomena: veering, locking and strong coupling effects. J. Acoust. Soc. Am. 131, 1015–1028 (doi:10.1121/1.3672647) 22352477 10.1121/1.3672647

[46] Vijayan K Woodhouse J 2014 Shock amplification, curve veering and the role of damping. J. Sound Vib. 333, 1379–1389 (doi:10.1016/j.jsv.2013.10.037)

